# Transcriptional response of OmpC and OmpF in *Escherichia coli* against differential gradient of carbapenem stress

**DOI:** 10.1186/s13104-019-4177-4

**Published:** 2019-03-14

**Authors:** Shiela Chetri, Melson Singha, Deepshikha Bhowmik, Kathakali Nath, Debadatta Dhar Chanda, Atanu Chakravarty, Amitabha Bhattacharjee

**Affiliations:** 1 Department of Microbiology, Assam University, Silchar, Assam India; 2 Department of Microbiology, Silchar Medical College and Hospital, Silchar, Assam India

**Keywords:** OmpF, OmpC, micF, Carbapenem, *Escherichia coli*, Real time PCR

## Abstract

**Objective:**

This study was designed to investigate the transcriptional response of OmpF and OmpC along with an antisense RNA, MicF under concentration gradient carbapenem exposure.

**Result:**

An elevation in the expression of OmpF gene under concentration gradient imipenem stress from a particular concentration was observed. For OmpC gene a significant decrease in the expression was noticed under concentration gradient imipenem and meropenem stress. The study showed reduction in the expression of OmpC gene against imipenem and meropenem possibly preventing the entry of carbapenem antibiotic inside the cell indicating a possible role in carbapenem resistance.

**Electronic supplementary material:**

The online version of this article (10.1186/s13104-019-4177-4) contains supplementary material, which is available to authorized users.

## Introduction

OmpC and OmpF are two major outer membrane proteins in *E. coli* which serve as a barrier for antibiotics and other toxic agents entering inside the cell [1]. It is reported that sub lethal dose of antibiotics has protective role in bacterial cell against wide range of antimicrobials [2] and down regulation of porin genes are responsible in nonspecific resistance. OmpC and OmpF are known to be involved in non-specific solute transport and also it was reported that multidrug resistant *E. coli* had lower level of OmpC expression [3]. Also, previous reports suggest that OmpC and OmpF share reciprocal relationship [4].

## Main text

### Methodology

#### Bacterial strains

A total of 96 consecutive, non-duplicates, *Escherichia coli* clinical isolates resistant to at least one of the carbapenem antibiotic were collected from patients attended the clinic or admitted between August 2016 and July 2017 in Silchar Medical College and Hospital, Silchar, India.

#### Antibiotic susceptibility testing

A preliminary antibiotic susceptibility testing was performed to select the study isolates. Susceptibility was performed by Kirby Bauer method against different antibiotics viz, ciprofloxacin (5 μg),amikacin (30 μg),cefepime (30 μg), aztreonam (30 μg), ceftriaxone (30 μg), co-trimoxazole (25 μg), ceftazidime (30 μg), levofloxacin (5 μg) gentamicin (10 μg), carbenicillin (10 μg), ceftazidime (30 μg) and piperacillin-tazobactam (100/10 μg) (Hi-media, Mumbai, India) and results were interpreted as per Clinical and laboratory standards institute guidelines [5]. *E. coli* ATCC 25922 was used as control.

#### Minimal inhibitory concentration (MIC) assay

As the study intends to investigate the OmpC and OmpF transcriptional expression under carbapenem stress, the minimum inhibitory concentration of the test isolates was determined against imipenem, meropenem and ertapenem by agar broth dilution method at varied concentration ranging from 0.125 to 512 µg/ml and the results were interpreted as per CLSI guidelines [5].

#### Detection of efflux pump mediated carbapenem resistance using an inhibitor

Porin loss/mutation and increased efflux pump activity are major contributor of innate resistance mechanism. Therefore, the efflux pump mediated carbapenem resistance was assessed using an inhibitor. This test was carried out for all the test isolates with meropenem (10 µg, Himedia, Mumbai) with and without an efflux pump inhibitor carbonyl cyanide m-chlorophenylhyrazone (12.5 µM), [Himedia, Mumbai, India]. A difference between zone of inhibition of ≥ 5 mm with the inhibitor and the carbapenems alone confirms to be having efflux pump activity. Ethidium bromide was taken as control substrate for the efflux pump activity [6]. Disc with only CCCP was used to rule out any activity of the agent alone.

#### Detection of carbapenemases

To investigate the presence of carbapenemase activity among the selected isolates Modified Hodge test was performed. Further, polymerase chain reaction (PCR) assay was carried out in a 96 well thermal cycler (Applied Biosystems) in order to detect various carbapenemase-encoding genes which included *bla*_KPC_*, bla*_IMP_, *bla*_VIM_, *bla*_NDM_, *bla*_OXA-23_, *bla*_OXA-48_ and *bla*_OXA-58_ [7–10]. This experiment was performed to rule out the role of any acquired mechanism in carbapenem resistance among study isolates.

#### Analysis of efflux pump component acrA gene expression

To detect the presence of any isolates with overexpressed acrAB-tolC system, the non carbapenemase producers were further selected and subjected to quantitative Real Time PCR to examine the expressional level of the efflux pump gene acrA under normal condition. The total RNA was isolated using QIAGEN Rneasy Mini Kit (QIAGEN, Germany) following manufacturer’s instructions. Estimation of the isolated RNA was performed using Picodrop (Pico200, Cambridge, UK) and further cDNA was synthesized using prepared mRNA as template via Qiagen Reverse Transcription Kit (QIAGEN, Germany). Quantitative Real-time PCR of the cDNA prepared was done using power Sybrgreen PCR master mix reagents kit (Applied Biosystems, Austin, USA) in StepOnePlus quantitative Real Time-PCR (Applied Biosystems, USA) in triplicates using specific primers-acrA (F): 5′CTCTCAGGCAGCTTAGCCCTAA3′, acrA (R): 5′TGCAGAGGTTCAGTTTTGACTGTT3′ [11]. *E. coli* ATCC 25922 and *RpslE* was used as quality control and endogenous control.

#### Transcriptional expression of OmpF and OmpC and an antisense RNA gene micF gene

The overnight cultures of *E. coli* isolates on Luria Broth (Hi-media, Mumbai, India) were centrifuged and subjected to total RNA isolation using QIAGEN Rneasy Mini Kit (QIAGEN, Germany) according to manufacturer’s guidelines. The isolated RNA was then estimated with the use of Picodrop (Pico200, Cambridge, UK) and further synthesis of cDNA was done using Qiagen Reverse Transcription Kit (QIAGEN, Germany). Quantitative Real-time PCR of the cDNA prepared was performed by using power Sybrgreen PCR master mix reagents kit (Applied Biosystems, Austin, USA). Analysis of the synthesized cDNA levels was carried out in triplicate in StepOnePlus quantitative Real Time-PCR (Applied Biosystems, USA) using specific primers (Additional file 1: Table S1). *E. coli* ATCC 25922 was used as a reference for the analysis of relative fold changes in gene expression.

#### Transcriptional expression of OmpF and OmpC porin genes and MicF gene under concentration gradient carbapenem stress

To investigate the role of porin genes OmpF and OmpC during carbapenem stress the isolates were grown on LB broth containing 0.25 µg/ml, 0.5 µg/ml, 1 µg/ml, 2 µg/ml and 4 µg/ml of imipenem, meropenem and ertapenem individually. The culture was incubated for 16 h till late log phase. The mRNA was isolated immediately and reverse transcribed into cDNA. Quantitative Real Time PCR was performed to assess the transcriptional response of OmpF, OmpC and MicF against concentration gradient carbapenem stress. All the protocols were followed as described earlier in the text.

### Statistical analysis

The relative expression differences of porin genes OmpF and OmpC along with an antisense RNA gene, MicF was compared with their respective wild type strain under concentration gradient carbapenem stress between samples were determined with the help of one-way ANOVA followed by Tukey–Kramer (Tukey’s W) multiple comparison test. Differences were considered statistically significant at both 5% and 1% level when p < 0.05. SPSS version 17.0 was used for statistical analysis.

## Result

**Fig. 1:**
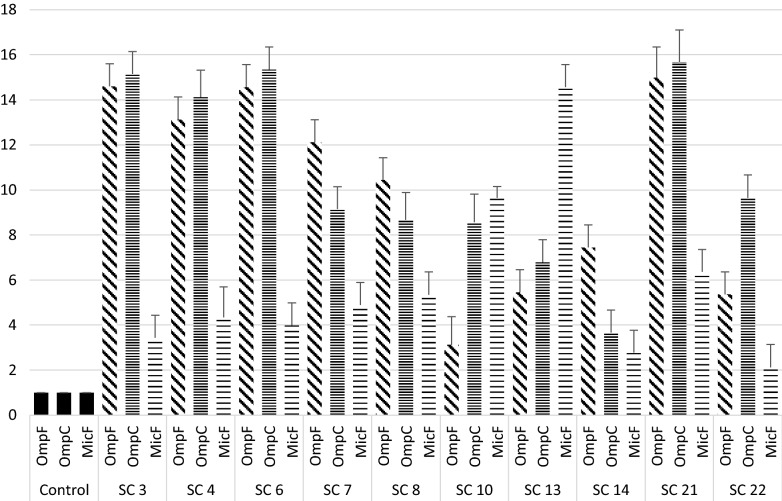
Expression of OmpF, OmpC and micF gene under normal condition (without stress) relative to *E. coli* ATCC 25922

**Fig. 2:**
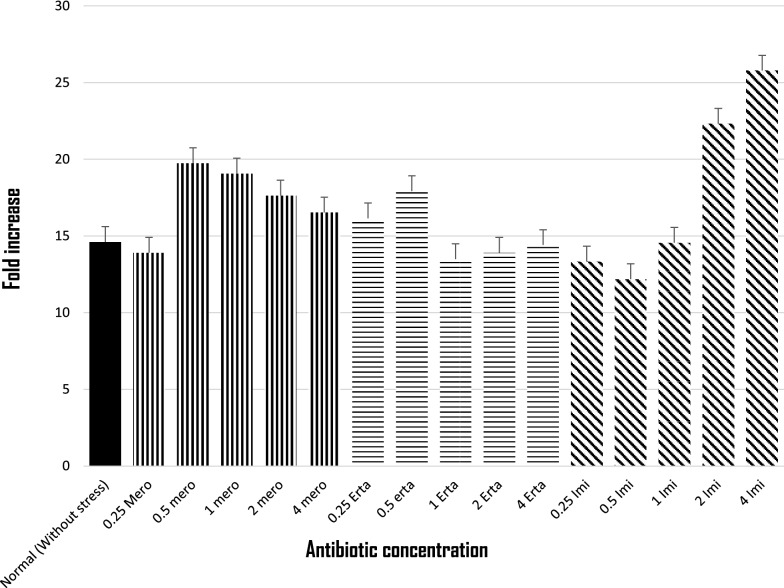
Expression of OmpF gene under concentration gradient carbapenem stress relative to the normal one without stress

**Fig. 3:**
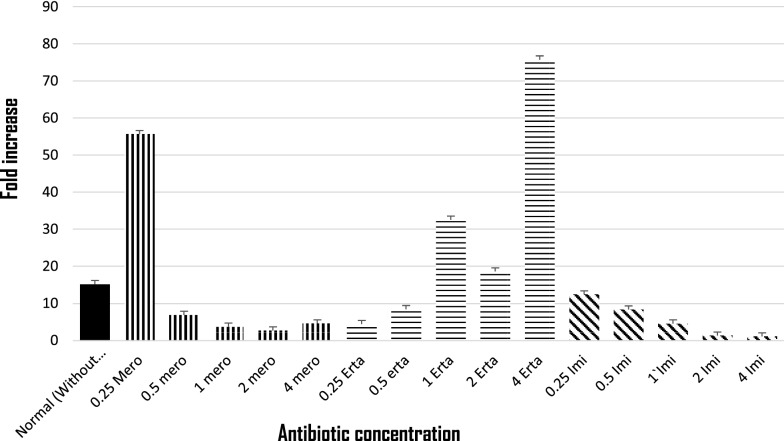
Expression of OmpC gene under concentration gradient carbapenem stress relative to the normal one without stress

Among 96 carbapenem non-susceptible and carbapenemase non-producer *E. coli*, 39.5% (38/96) were selected based on their resistance towards at least one of the carbapenems tested. Of which 57.89% (22/38) isolates showed efflux pump positive activity phenotypically when tested with meropenem along with an efflux pump inhibitor CCCP. Antimicrobial susceptibility of the isolates showed highest susceptibility towards amikacin (86.36%) followed by cefepime (59.09%) and co-trimoxazole (40.90%). MIC result revealed that more than half of the isolates were found to be above the break point against ertapenem (90.9%), followed by imipenem (77.2%) and meropenem (59.0%) (Additional file 2: Table S2). Further, the transcriptional expression of acrA exhibited over expression of acrA gene in 12 isolates and 10 isolates showed low level of expression (Additional file 3: Figure S1) when compared with *E. coli* ATCC 25922. Isolates with reduced expression of acrA gene were selected and further studied for mRNA transcript of the porin genes OmpF and OmpC along with MicF (Fig. 1), of which all the three genes exhibited higher expression. However, when the expressional level was compared with the stressed condition, expression of OmpF gene was elevated against concentration gradient imipenem stress from 0.5 µg/ml, whereas; under meropenem stress a steady decrease in the expression was recorded (Fig. 2). Ertapenem exposures showed random transcriptional level (Fig. 2). For OmpC gene a significant decrease in the expression was noticed under concentration gradient imipenem and meropenem stress (Fig. 3). While against ertapenem stress an asymmetric pattern of expression was observed (Fig. 3). In case of MicF a steady decrease in the expression after meropenem exposure was observed and ertapenem and imipenem stress showed an arbitrary pattern in the expression (Additional file 4: Figure S2).

## Discussion

This study deals with the outer-membrane porin genes OmpF, OmpC and MicF gene which perhaps has a role in developing resistance towards carbapenems. As reported by Nikaido and Vaara, the expression of OmpF gene is reduced in high osmolarity condition whereas, for OmpC the same environment was found to be favorable [12]. However, efflux pump gene AcrB has also been reported to repress the expression of the porin OmpF gene [13, 14]. In this study carbapenem resistant *E. coli* isolates were studied of which decrease in the expression of OmpF and OmpC genes were observed in AcrAB overexpressed isolates which is in agreement with previous studies [15–17]. In a study done by Yigit et al., in *Enterobacter* strain 810, alteration in the porin genes OmpF and OmpC resulted in imipenem resistance along with reduced susceptibility towards meropenem and cefepime [18]. After, exposure to sub inhibitory concentration of imipenem down regulation of OmpC gene was also observed which is in support of the present study. However, it was observed under imipenem stress an increase in the expression of OmpF gene was observed which has not been reported earlier. The expression level of OmpF is also affected by a small antisense RNA, MicF which inhibits the expression of OmpF gene [19]. However, in the present study meropenem stress reduced the expression of OmpF and micF as well, indicating that there may be other factors responsible for the down regulation of OmpF against carbapenem exposure. Therefore, it is probably the major porin that prevents entry of carbapenem inside the cell which calls for further investigation.

## Limitation

This study warrants a proteomic approach to analyze the expression of porin genes at translational level.

## Additional files

**Additional file 1: Table S1.** Oligonucleotides used in the study.

**Additional file 2: Table S2.** Minimum inhibitory concentration range of carbapenems tested.

**Additional file 3: Figure S1.** Expression of acrA gene under normal condition (without stress) relative to *Escherichia coli* ATCC 25922.

**Additional file 4: Figure S2.** Expression of MicF gene under concentration gradient carbapenem stress relative to normal one without stress.

## Abbreviations

OmpF: outer membrane protein F
OmpC: outer membrane protein C
CCCP: carbonyl cyanide m-chlorophenylhydrazone
