# Supplementary material for: Transcriptional response of OmpC and OmpF in Escherichia coli against differential gradient of carbapenem stress
Source: BMC Res Notes. 2019 Mar 14;12:138. doi: 10.1186/s13104-019-4177-4 (PMC6419367; doi:10.1186/s13104-019-4177-4)
Supplement: Supplementary file 1 — Additional file 1: Table S1. Oligonucleotides used in the study. [file 13104_2019_4177_MOESM1_ESM.docx]

**Additional file 1: Table S1**: Oligonucleotides used in the study

| **Sl No.** | **Primer Pair** | **Targets** | **Sequence (5́→3́)** | **Product Size (bp)** | **Reference** |
| --- | --- | --- | --- | --- | --- |
| 1 | OmpF F  OmpF R | OmpF | AAGTAGTAGGTTGCGCCCAC  AGTTCGATTTCGGTCTGCGT | 118 | This study |
| 2 | OmpC F  OmpC R | OmpC | ATTCTGGCAGTACGTCGGTC  AAACAACTCCTGGACCCGTG | 125 | This study |
| 3 | MicF F  MicF R | MicF | TCATCATTAACTTTATTTATTACCG  GCATCCGGTTGAAATAGG | 70 | This study |
