# Supplementary material for: Transcriptional response of OmpC and OmpF in Escherichia coli against differential gradient of carbapenem stress
Source: BMC Res Notes. 2019 Mar 14;12:138. doi: 10.1186/s13104-019-4177-4 (PMC6419367; doi:10.1186/s13104-019-4177-4)
Supplement: Supplementary file 2 — Additional file 2: Table S2. Minimum inhibitory concentration range of carbapenems tested. [file 13104_2019_4177_MOESM2_ESM.docx]

**Additional file 2: Table S2**: **Minimum inhibitory concentration Range of carbapenems tested.**

| **Antibiotics** | **MIC range examined**  **(µg ml^-1^)** | | | | | | | | | | | | | | **No. (%) of isolates above the breakpoint**  **(N= 22)** |
| --- | --- | --- | --- | --- | --- | --- | --- | --- | --- | --- | --- | --- | --- | --- | --- |
|  | < 0.25 | 0.25 | 0.5 | 1 | 2 | 4 | 8 | 16 | 32 | 64 | 128 | 256 | 512 | > 512 |  |
| Meropenem | 3 | 2 | 2 | 2 | 1 | 1 | 1 | 4 | 2 | 1 | 1 | 1 | - | 1 | 59.0(n=13) |
| Ertapenem | 1 | - | 1 | 4 | 1 | 3 | - | - | 3 | 2 | 3 | 1 | 2 | 1 | 90.9(n=20) |
| Imipenem | 1 | 1 | 2 | 1 | 1 | 1 | 4 | 3 | 1 | 2 | 3 | 1 | - | 1 | 77.2(n=17) |

**N=Total number of isolates, n= Number of isolates above the break point**
