# Supplementary material for: Transcriptional response of OmpC and OmpF in Escherichia coli against differential gradient of carbapenem stress
Source: BMC Res Notes. 2019 Mar 14;12:138. doi: 10.1186/s13104-019-4177-4 (PMC6419367; doi:10.1186/s13104-019-4177-4)
Supplement: Supplementary file 3 — Additional file 3: Figure S1. Expression of acrA gene under normal condition (without stress) relative to Escherichia coli ATCC 25922. [file 13104_2019_4177_MOESM3_ESM.docx]

**1=Indicates the control used in the study (*E. coli* ATCC 25922), 2-17=Test isolates**

**Additional file 3: Figure S1: Expression of acrA gene under normal condition (without stress) relative to *Escherichia coli* ATCC 25922**
