# Supplementary material for: Transcriptional response of OmpC and OmpF in Escherichia coli against differential gradient of carbapenem stress
Source: BMC Res Notes. 2019 Mar 14;12:138. doi: 10.1186/s13104-019-4177-4 (PMC6419367; doi:10.1186/s13104-019-4177-4)
Supplement: Supplementary file 4 — Additional file 4: Figure S2. Expression of MicF gene under concentration gradient carbapenem stress relative to normal one without stress. [file 13104_2019_4177_MOESM4_ESM.docx]

**Additional file 4: Figure S2: Expression of MicF gene under concentration gradient carbapenem stress relative to normal one without stress.**
